# Microglia and brain macrophages are differentially associated with tumor necrosis in glioblastoma: A link to tumor progression

**DOI:** 10.32604/or.2024.056436

**Published:** 2025-03-19

**Authors:** CHRISTINA LOH, YUQI ZHENG, ISLAM ALZOUBI, KIMBERLEY L. ALEXANDER, MAGGIE LEE, WEI-DONG CAI, YANG SONG, KERRIE MCDONALD, ANNA K. NOWAK, RICHARD B. BANATI, MANUEL B. GRAEBER

**Affiliations:** 1 Ken Parker Brain Tumor Research Laboratories, Brain and Mind Centre, Faculty of Medicine and Health, The University of Sydney, Sydney, NSW 2050, Australia; 2 School of Computer Science, The University of Sydney, Sydney, NSW 2008, Australia; 3 Neurosurgery Department, Chris O’Brien Lifehouse, Camperdown, NSW 2050, Australia; 4 Department of Neuropathology, Royal Prince Alfred Hospital and Brain and Mind Centre, Faculty of Medicine and Health, The University of Sydney, Sydney, NSW 2006, Australia; 5 School of Computer Science and Engineering, University of New South Wales, Sydney, NSW 2052, Australia; 6 Brain Cancer Consultancy, Sydney, NSW 2040, Australia; 7 Medical School, University of Western Australia, Crawley Campus, Perth, WA 6009, Australia; 8 Faculty of Medicine and Health, The University of Sydney, Sydney, NSW 2050, Australia; 9 Santuario Accademico S. Giovanni D’Andorno, Casa Alpina ‘Principessa Laetitia’, Frazione Bele, Campiglia Cervo, 13812, Italy; 10 University of Sydney Association of Professors (USAP), University of Sydney, Sydney, NSW 2006, Australia

**Keywords:** Bone marrow-derived macrophages (BMDM), CD163, Glioblastoma/glioma stem cells (GSCs), IBA1, Microglia, Multimodal whole slide analysis, Tumor microenvironment

## Abstract

**Background:**

Microglia and brain macrophages contribute significantly to the tumor microenvironment in highly malignant glioblastoma where they are considered important drivers of tumor progression. A better understanding of the role of the brain macrophages present in glioblastoma appears crucial for improving therapeutic outcomes, especially in the context of novel immunotherapeutic approaches.

**Methods:**

We investigated the regulation of two well-established markers for microglia and brain macrophages, IBA1 and CD163, in relation to glioblastoma tumor necrosis using immunohistochemistry and modality fusion heatmaps of whole slide images obtained from adjacent tissue sections.

**Results:**

IBA1 and CD163 showed remarkable differences in relation to glioblastoma tumor necrosis. Generally, IBA1 immunoreactive cells were far less common in necrotic tissue areas than CD163-expressing cells. We also found extensive and frequently diffuse extracellular CD163 deposition, especially in hypocellular necrobiotic tumor regions where IBA1 was typically absent.

**Conclusions:**

Resident microglia seem more likely to be important for the diffuse infiltration of glioma cells in hypercellular tissue areas, whereas myeloid macrophages may be the main macrophage population in the wake of tumor necrosis. Since the necrotic niche with its interactions between microglia, brain macrophages, and glioblastoma/glioma stem cells is increasingly recognised as an important factor in tumor progression, further detailed studies of the macrophage populations in glioblastoma are warranted.

## Introduction

The microglia of the central nervous system (CNS) and bone-marrow-derived macrophages are phagocytes that under disease conditions typically eliminate cellular debris from tissue. It seems counter-intuitive therefore that they would not equally associate with tumor necrosis in glioblastoma. However, we have observed striking differences in the regulation of the microglia and brain macrophage markers, IBA1 and CD163, in relation to glioblastoma tumor necrosis, and in the distribution of non-cell bound macrophage antigen in the case of CD163. These disparities raise intriguing questions because microglia and brain macrophages are both considered to contribute to tumor progression and can be influenced by glioma cells to change their behavior. Moreover, necrosis, a characteristic feature of the glioblastoma tumor environment, has a pivotal role in glioblastoma progression [1,2], and this process is reinforced by the interaction of glioma stem cells (GSCs) with both microglia and macrophages [3]. Our present study aims to provide deeper insights into this complexity.

Tumor-associated macrophages (TAMs) are attracting increasing attention in glioblastoma research [4,5]. In the human brain, both resident microglia and invading bone marrow-derived macrophages (BMDM) contribute to the macrophage cell pool in glioblastoma. The literature on this topic is growing rapidly [6–8]. IBA1, the ionized calcium-binding adapter molecule 1, is expressed in normal resident microglia and plays a role in phagocytosis. It is strongly upregulated in activated microglial cells and has therefore become a widely used diagnostic marker for the assessment of microglial cells including in glioblastoma [9,10]. In contrast, CD163 is present on (BMDM) but typically absent from resident microglia and most activated microglial cells [11]. Interestingly, there is evidence of a correlation between the presence of CD163-expressing macrophages and more aggressive growth across various types of cancer suggesting that the presence of higher numbers of CD163 immunoreactive macrophages is indicative of a poor prognosis [12–15].

Necrosis is a histological hallmark of malignancy in glioma but the association of different activation states of microglia and brain macrophages with different stages and types of necrosis in glioma has not been studied in detail. We have utilized two well-established microglia and brain macrophage markers, IBA1 and CD163, in combination with computer-assisted diagnostic algorithms applied to whole slide images/scans (WSIs). This has enabled us to investigate the co-occurrence of microglia and brain macrophages within and around glioblastoma necrosis in a systematic manner and at a level of detail not possible before.

## Materials and Methods

### Case material

**Figure 1:**
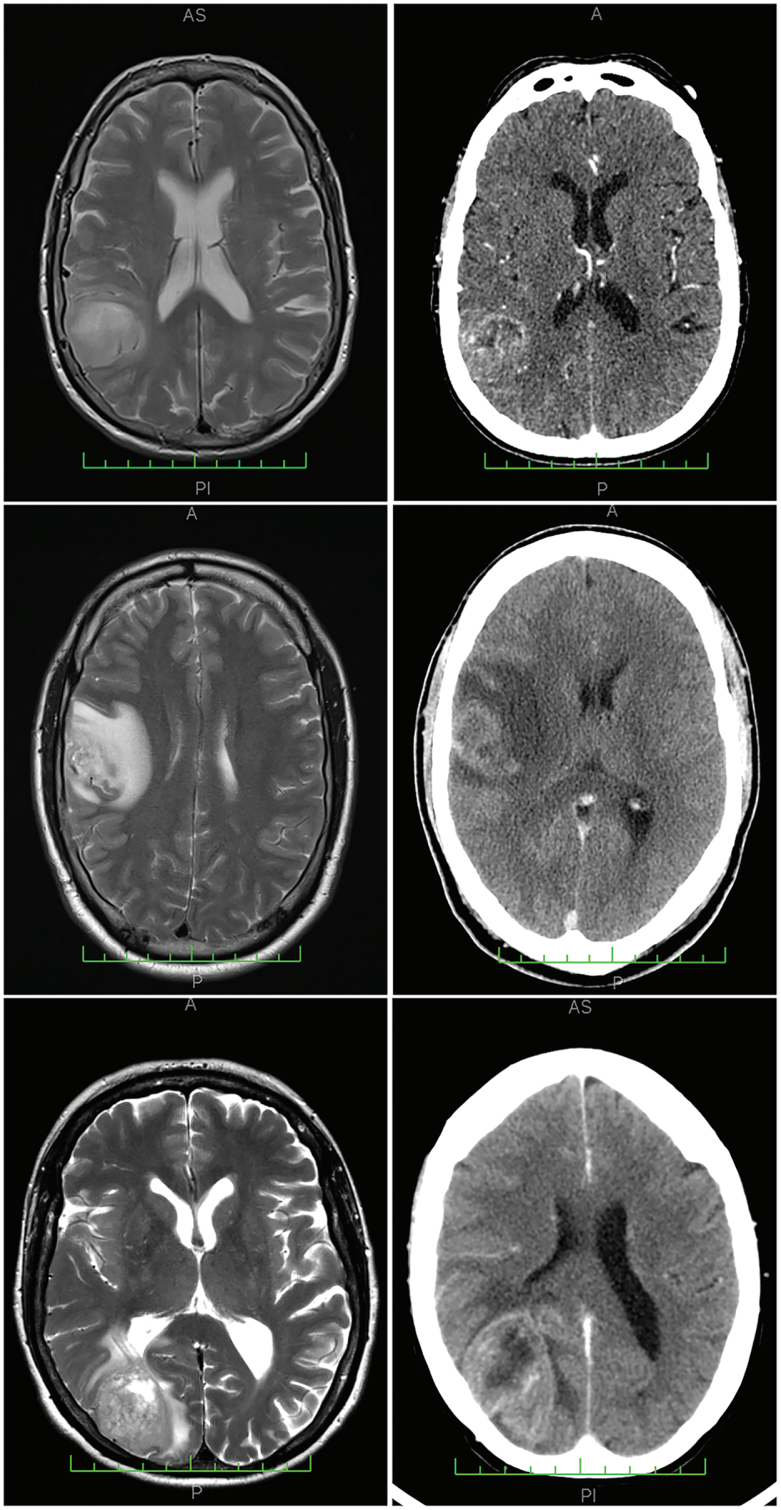
Neuroimaging results from three representative cases analyzed, with corresponding MRI (magnetic resonance imaging) scans on the left and CT (computerized tomography) scans on the right. The cases presented are: Case 25 (top row), Case 6 (center row), and Case 1 (bottom row), all from Cohort 1. Example histological findings for these cases can be found in Fig. 3.

This research involves the analysis of two distinct cohorts of human glioblastoma cases (Cohort 1 and Cohort 2, see Tables A1, A2). Cohort 1 of 34 WHO Grade 4 glioma samples used in this study was provided by the Australian Genomics and Clinical Outcomes of Glioma (AGOG) tissue bank (University of Sydney Human Ethics Committee Project number 2016/027). A second independent cohort of 25 cases was provided by the Sydney Brain Tumor Bank (Royal Prince Alfred Hospital Ethics Committee Project number 2019/ETH07282) and used for validation purposes. Paraffin sections were stained with H&E (Hematoxylin and Eosin) and scanned using an Olympus VS-120 scanner (VS120 Virtual Slide System, Olympus, Japan). The Department of Neuropathology at Royal Prince Alfred Hospital processed adjacent sections for immunohistochemistry. The well-established microglia and macrophage markers IBA1 and CD163 were processed according to manufacturers’ recommendations. Anti-CD163, clone 10D6 (Leica, Australia, cat no. CD163-L-CE), was used following antigen retrieval at 90°C for 30 min. Incubation was carried out at 1:200 for 60 min. Anti-IBA1 (Wako, Japan, cat no. 019-19741) was incubated at 1:1000 for 60 min following antigen retrieval. Whole-slide scans of the immunolabeled paraffin sections were obtained at 40× magnification. All cases had been originally diagnosed according to the fourth edition of the WHO classification for CNS tumors which was updated in 2021. This required the reclassification of some cases which were excluded. Thirty-three glioblastoma cases remained in the first cohort. Thus, there were no consequences for the conclusions of this study. The neuroimaging of cases was also reviewed (Fig. 1, Tables A1, A2). Survival statistics were obtained by applying a Kaplan Meier and Cox Proportional Hazards model. QuPath [16] was used for viewing WSIs.

### Computer analysis

To facilitate the analysis of WSIs of immunohistochemically stained slides, computer-assisted diagnostic (CAD) algorithms were used. To identify immunoreactive areas, the entire scan of each digital slide (WSI) was divided into patches of 16 × 16 pixels and the intensity level in each patch was measured. The intensity was greatest in areas with the strongest immunoreactive labeling, i.e., the presence of a brown color of the diaminobenzidine (DAB) peroxidase reaction product which indicates the location of the protein to be detected. An automatic image processing method was developed to extract the brown regions in the images. This was achieved by first using k-means clustering so that pixels of similar values were grouped. The number of clusters was then determined automatically. Subsequently, a threshold value was selected based on empirical observations of the images within the dataset. Clusters that were brown and darker than this threshold value were extracted as the brown regions of interest. Areas of high marker expression (marked red on the turquoise heatmap) could be identified after iteratively calculating the intensity average from the extracted patches. Thus, the strongest immunoreactivity of a pixel in our cropped images corresponds to the highest intensity value.

This approach allowed the detection of variations in the density of the respective marker immunoreactivity across different areas of the WSI simply by observing color changes. The approach is therefore analogous to what a human observer does, but it is performed with much greater precision and to completion for each slide. Image fusion then enabled the precise co-localization of pathomorphological features and immunohistochemical labeling results, increasing the amount of information obtained from biopsy specimens. A conditional method was employed for fusing images of sections. Sections were immunolabeled for IBA1 and CD163, and the color maps of corresponding pixels from CD163 and IBA1 input heatmaps were utilized to analyze the immunolabeled sections. If they were different, then the color white was used. Thus, by combining complementary information from multiple sources into a single image, medical image fusion can produce higher-quality content. Correlation analyses were employed to confirm differences in marker expression. Pixel color intensity analyses of IBA1 and CD163 heatmaps were carried out to perform detailed comparisons of immunoreactivity in adjacent slides. The correlation of IBA1 and CD163 immunolabeling was visualized in bimodal WSIs following image fusion of the corresponding predicted heatmaps.

## Results

### Differential expression of the microglia/brain macrophage markers, IBA1 and CD163

The two microglia/macrophage markers employed in this study, IBA1 and CD163, showed strikingly different patterns of expression between glioblastoma cases. There was also only a limited match between IBA1 and CD163 immunoreactivity in numerous areas within the same tumor. Thus, while both IBA1 and CD163 are markers used to identify microglia and macrophages, there was no strong correlation or co-localization between their expression patterns in many regions of the glioblastoma samples studied although, importantly, an association was observable in other and sometimes adjacent tumor regions making this analysis quite complex and difficult for the human eye. This may explain why it has not been undertaken previously. Remnants of histologically normal-appearing brain tissue showed the typical IBA1 labeling of ramified resident microglia and occasional perivascular macrophages whereas CD163 was typically absent from histologically normal-appearing brain tissue with the exception of perivascular macrophages. Since IBA1 is known to label both phagocytic microglia and full-blown brain macrophages in brain pathologies, our finding of a strikingly different pattern of expression of IBA1 and CD163 in association with tumor necrosis was surprising.

**Figure 2:**
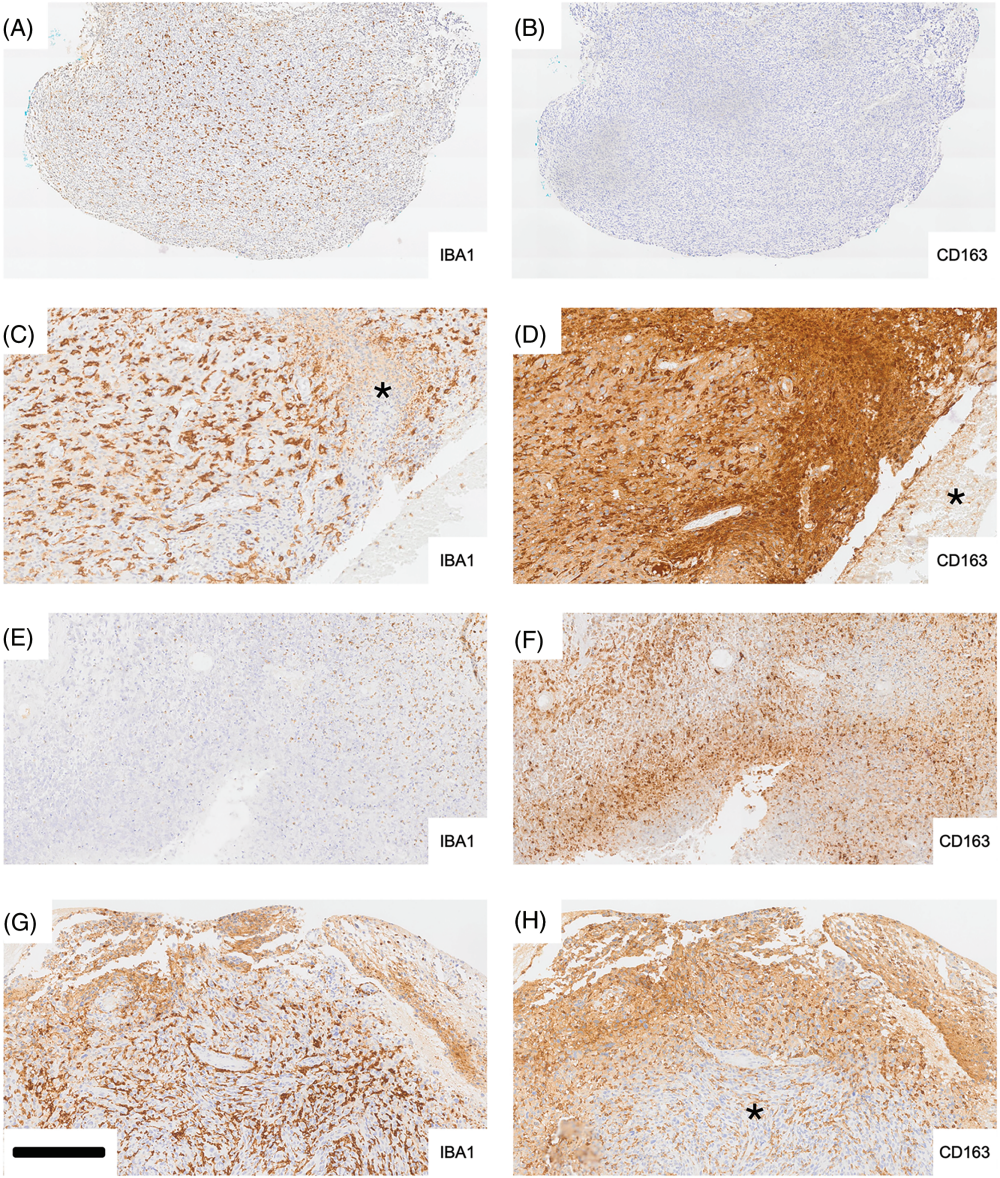
Differential expression of IBA1 and CD163 in glioblastoma (adjacent tissue sections). Iba-1 labeling (A, C, E, G) is typically cellular whereas staining for CD163 (B, D, F, H) frequently shows extracellular deposition of the peroxidase reaction product, diaminobenzidine (DAB) (D, F, H) as well. In cases where we found differential expression, CD163 immunoreactivity could be very strong in some tissue areas where Iba-1 was weak in the corresponding part of the adjacent section (e.g., the area marked by the asterisk in C). The area in C shows a high density of nuclei probably representing tumor cells. However, other tissue areas (asterisk in D and corresponding area in C) could be almost completely devoid of staining. E and F represent another pair of adjacent tissue areas showing discrepant expression for the two microglia/macrophage markers, Iba-1 and CD163. In E, Iba-1 immunoreactivity is weak and very few recognizable microglia/macrophages are present. F demonstrates CD163 staining especially over tissue displaying reduced tissue integrity (necrosis?). In contrast, in H immunoreactivity for CD163 is much weaker than that of Iba-1 (G) in the corresponding tumor area (asterisk in H) but CD163 labeling is stronger than that for Iba-1 towards the upper tumor margin (necrosis?) Scale bar: 500 µm for A and B, 200 µm for C to H.

Fig. 2 illustrates differences in expression between IBA1 and CD163 in glioblastoma at lower magnification (Fig. 2A,B). IBA1 labeling is confined to cells whereas CD163 immunoreactivity is also and not infrequently found extracellularly (e.g., Fig. 2D). There were tumor areas where IBA1 and CD163 were strongly and widely expressed throughout the tissue (Fig. 2C,D). However, in some tissue areas of the same section, CD163 immunoreactivity could be very low and the same was true for IBA1 expression in different areas (asterisks in Fig. 2C,D). CD163 immunoreactivity in tumor tissue in the absence of IBA1 upregulation was a rare finding and potentially related to incipient necrosis based on morphological criteria. For instance, such intense CD163 expression in a band-like fashion is shown in Fig. 2F (compared to 2E). In cases where we found differential expression, CD163 immunoreactivity could be very strong in some tissue areas where IBA1 was weak in the corresponding part of the adjacent section, e.g., the area marked by the asterisk in Fig. 2C. This area in Fig. 2C shows a high density of nuclei probably representing tumor cells. However, other tissue areas (asterisk in Fig. 2D and the corresponding area in Fig. 2C) could be almost completely devoid of staining. Fig. 2E,F represents another pair of adjacent tissue areas showing discrepant expression for the two microglia/macrophage markers, IBA1 and CD163. In Fig. 2E, IBA1 immunoreactivity is weak and very few recognisable microglia/macrophages are present. Fig. 2F demonstrates CD163 staining especially over tissue displaying reduced tissue integrity (necrosis?). In contrast, in Fig. 2H immunoreactivity for CD163 is much weaker than that of IBA1 (Fig. 2G) in the corresponding tumor area (asterisk in Fig. 2H) but CD163 labeling is stronger than that for IBA1 towards the upper tumor margin (necrosis?).

These results demonstrate great intra-tumoral heterogeneity in the expression of IBA1 and CD163 in glioblastoma. CD163 labeling of large tissue areas could be observed in a number of biopsy samples. The latter is never seen in histologically normal brain tissue.

### Relationship of IBA1 and CD163 expression to tumor necrosis

**Figure 3:**
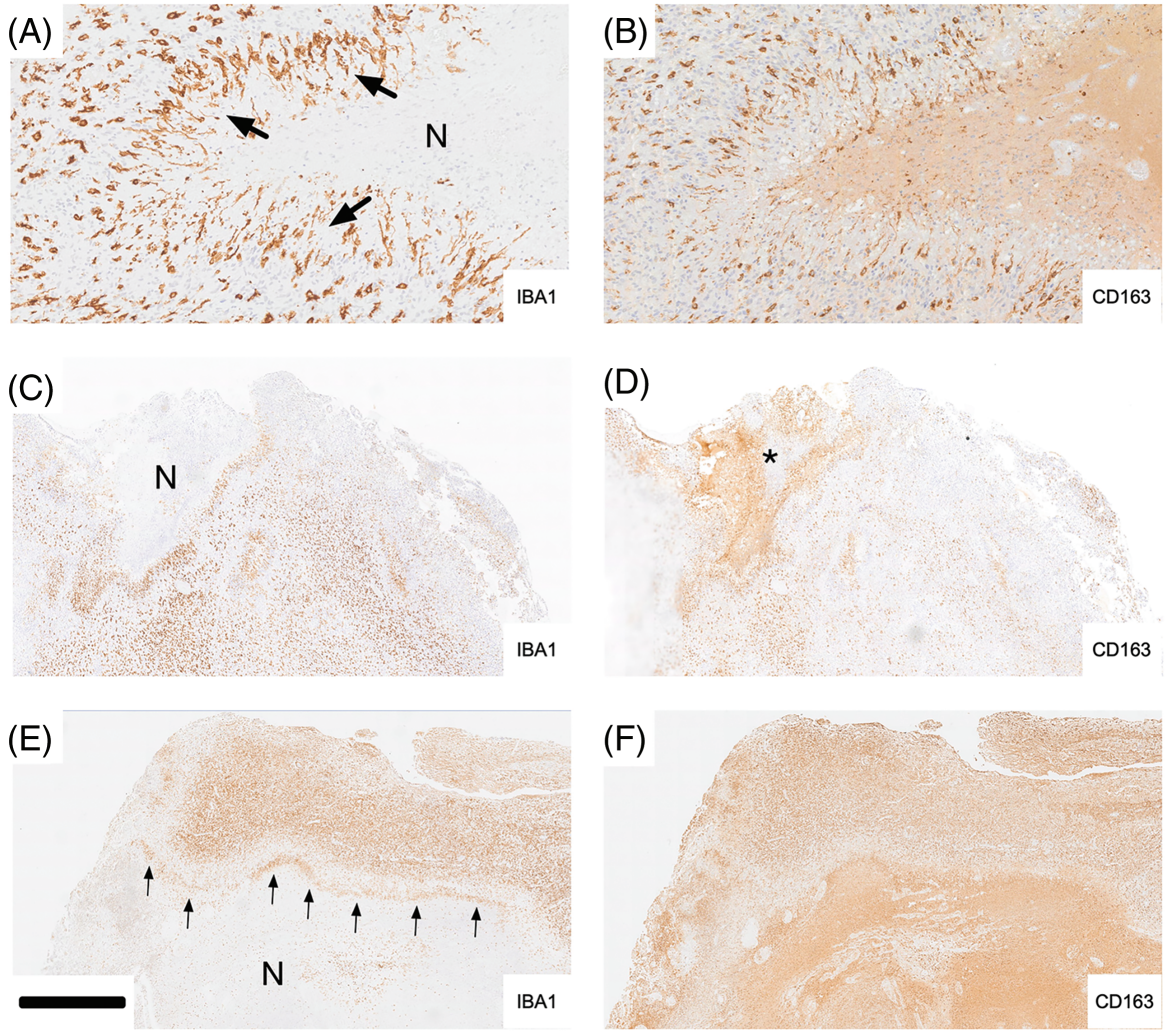
Examples of tumor necrosis are shown in (A–F). Iba-1 labeling is illustrated in the left column, and CD163 is on the right. In A, palisading microglia/macrophages (arrows) are strongly labeled for Iba-1 while expression for CD163 in the corresponding adjacent tumor area is much weaker but discernible on some palisading cells (B). It is noteworthy that the hypocellular part of the necrosis is strongly CD163 positive (staining is largely extracellular). C, A rim of Iba-1 immunoreactivity consisting of palisading microglia/macrophages surrounds a necrotic tissue area (corresponding asterisk in D) which is immunonegative for Iba-1 (C) but marked strongly for CD163 (D). E and F again illustrate significant differences between Iba-1 and CD163 expression. Iba-1 is expressed mainly in the upper half of what likely represents a cortical gyrus that is infiltrated by tumor cells. Palisading Iba-1 positive microglia/macrophages (cf. A and B) form a demarcation line (“rim”, arrows) above the necrotic tissue area which is largely devoid of Iba-1 staining but shows strong CD163 immunoreactivity in F. There is some accentuated CD163 staining at the rim. However, CD163 labeling is largely diffuse and most pronounced in the hypocellular tissue area (F). Scale bar: 200 µm in A, B; 1 mm in C-F. A and B are taken from Case 25, Fig. 3C and D are taken from Case 1, and E and F are taken from Case 6. N, necrotic core.

IBA1 staining of glioblastoma necrosis, especially of typical pseudo-palisading necrosis, highlighted bands of labeling (e.g., arrows in Fig. 3A,E) reflecting the presence of microglia/brain macrophages demarcating necrotic from viable tissue. In Fig. 3A, palisading microglia/macrophages (arrows) are strongly labeled for IBA1 while expression for CD163 in the corresponding adjacent tumor area is much weaker but discernible on some palisading cells (Fig. 3B). It is noteworthy that the hypocellular part of the necrosis is strongly CD163 positive (staining is largely extracellular). In Fig. 3C, a rim of IBA1 immunoreactivity consisting of palisading microglia/macrophages surrounds a necrotic tissue area (corresponding asterisk in Fig. 3D) which is immunonegative for IBA1 (Fig. 3C) but marked strongly for CD163 (Fig. 3D). Fig. 3E,F again illustrates significant differences between IBA1 and CD163 expression. IBA1 is expressed mainly in the upper half of what likely represents a cortical gyrus that is infiltrated by tumor cells. Palisading IBA1 positive microglia/macrophages (cf. Fig. 3A,B) form a demarcation line (“rim”, arrows) above the necrotic tissue area which is largely devoid of IBA1 staining but shows strong CD163 immunoreactivity in Fig. 3F. There is some accentuated CD163 staining at the rim. However, CD163 labeling is largely diffuse and most pronounced in the hypocellular tissue area (Fig. 3F).

**Figure 4:**
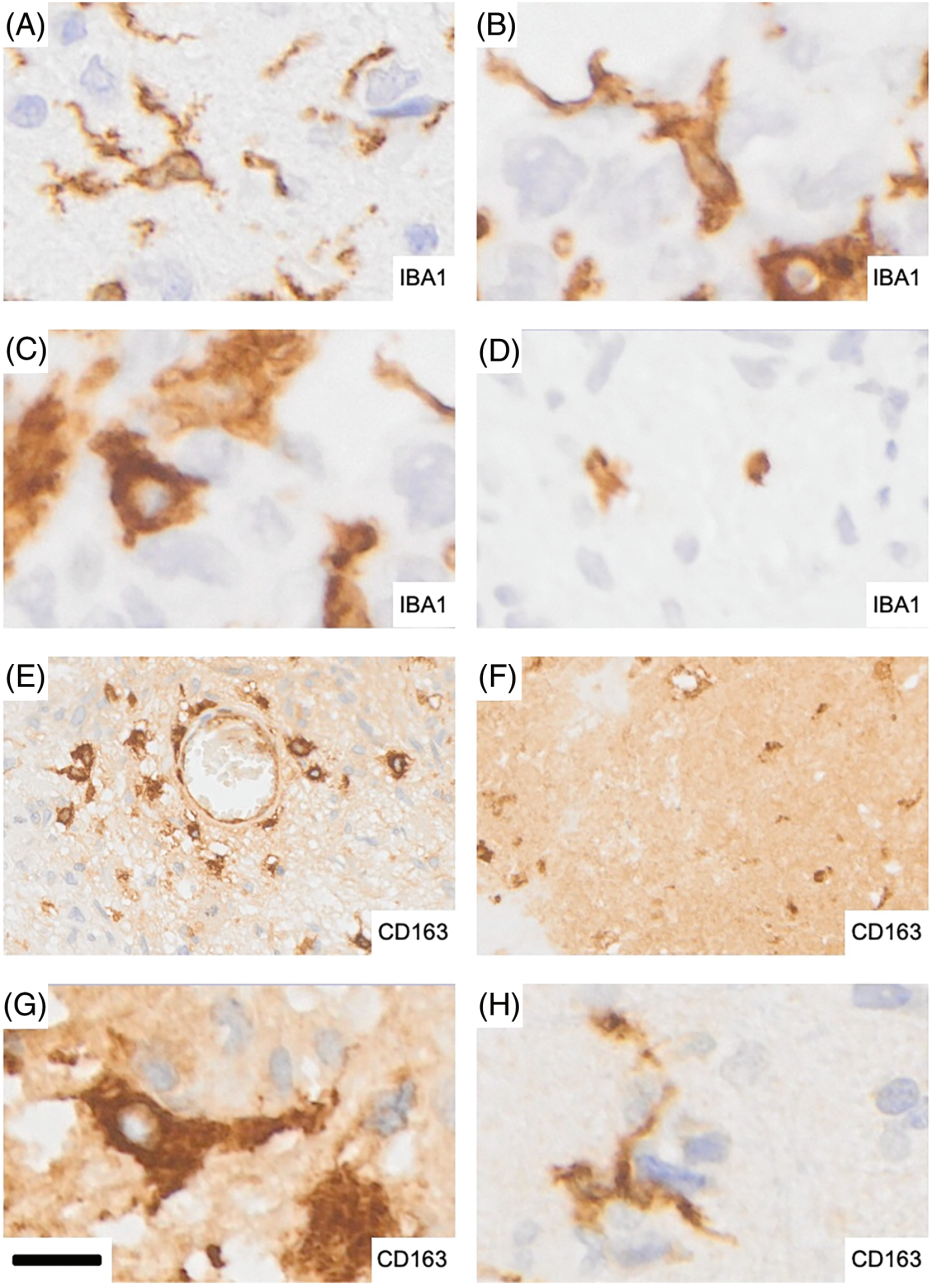
Representative microglia and macrophage morphologies in glioblastoma. (A) Ramified microglia showing expression of IBA1. (B) Strongly activated microglia display more intensely stained, stouter cell processes. (C) Rounder, macrophage-like microglia with strong IBA1 expression that are largely devoid of processes. (D) Isolated remnants of microglial cells with residual IBA1 immunoreactivity in a necrotic tumor area. (E) Perivascular extra- and intraparenchymal macrophages which are strongly CD163 positive. The distribution shown is reminiscent of recent infiltration from the bloodstream as seen in experimental animal studies. (F) Strong and largely diffuse immunoreactivity for CD163 in a necrotic tumor area: the majority of the staining is found extracellularly. (G) CD163 expressing macrophages. (H) A microglial cell expressing CD163. Scale bar: 12 µm for A–D and G–H and 40 µm for E–F.

Larger tumor necroses were characteristically devoid of cellular IBA1 labeling with the occasional exception of what appeared to be remnants of microglia cell bodies and/or their processes (Fig. 4D). In contrast, necrotic tumor areas were typically strongly and diffusely CD163 positive. Thus, necrosis showed the strongest extracellular CD163 labeling. Within pseudo-palisades, the occurrence of elongated IBA1 immunoreactive microglia was frequently matched by CD163 labeling of similar cells in adjacent sections but CD163 labeling in this location was weak in comparison and less widespread than IBA1 labeling. However, CD163 immunoreactivity was not always present where IBA1 expression was absent from necrotic brain tissue again attesting to the complexity of both labeling patterns.

Examples of tumor necroses are shown in Fig. 3. In Fig. 3A, “palisading microglia/macrophages” (arrows) are strongly labeled for IBA1 while expression of CD163 in the corresponding adjacent tumor tissue is faint (Fig. 3B). The hypocellular part of the necrosis is highly CD163 positive and the vast majority of labeling is extracellular. Intact appearing CD163 expressing macrophages which may be recently blood-derived (Fig. 4E) could be one source of the large amounts of CD163 which is deposited in many but not all areas of glioblastoma necrosis (Fig. 3D).

### Phenotypes of IBA1 and CD163 expressing cells

IBA1 labels highly ramified microglia and perivascular cells (macrophages) in normal brain tissue which is rarely found in tumor biopsies. In addition, even histologically normal-appearing brain tissue in a tumor’s vicinity cannot be considered completely unaffected. This becomes visible in sensitive immunohistochemical stains of pathologically altered brain tissue such as IBA1 where activated microglia phenotypes are detected that are characterized by shorter, stouter cell processes that less ramified and express increased IBA1 immunoreactivity (Fig. 4A,B). Consequently, the population of immunoreactive cells we encountered in glioblastoma encompassed a wide morphological spectrum ranging from still-ramified cells to plump rounded macrophages (Fig. 4C,G) depending on the pathological state of the tissues as shown in Fig. 4. Round, macrophage-like microglia with strong IBA1 expression were largely devoid of cell processes (Fig. 4C). Fig. 4E illustrates strongly labeled CD163 expressing brain macrophages within brain tissue around a blood vessel and the cellular density “gradient” displayed by them may be a reflection of their recent origin from bone marrow which can be demonstrated experimentally in animal studies. Strong and largely diffuse immunoreactivity for CD163 was typically present in necrotic tumor areas where most of the staining was found extracellularly (Fig. 4F). It should be mentioned that occasional ramified microglial cells can also exhibit CD163 expression in glioblastoma and other diseases (Fig. 4H).

### Simultaneous mapping of IBA1 and CD163 expression by means of image fusion

**Figure 5:**
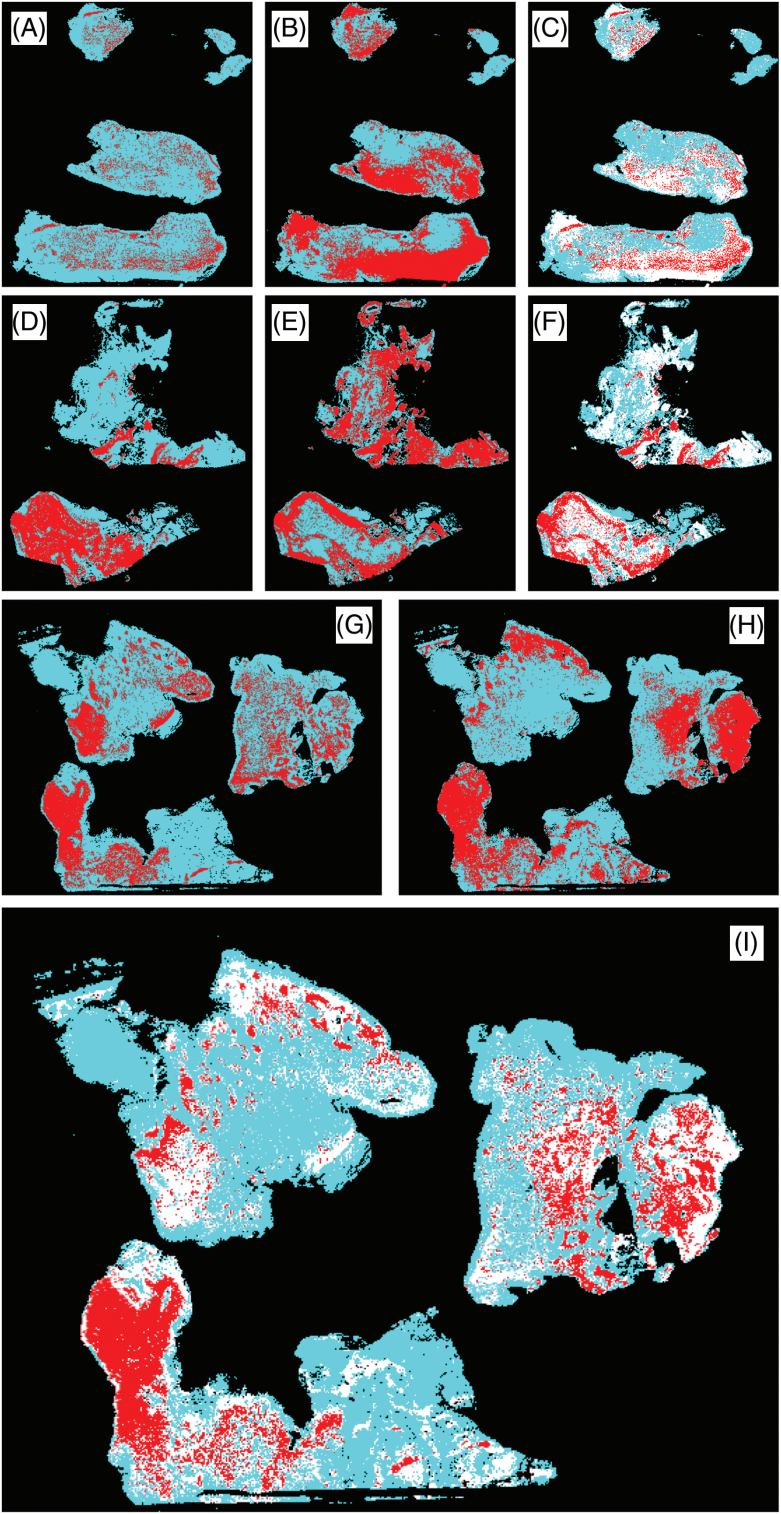
WSI fusion heatmaps (C, F, I) demonstrating the differential expression of IBA1 (A, D, G) and CD163 (B, E, H). Areas of overlap are shown in red and discrepant areas in white in the fusion heat maps. There are large areas of CD163 immunoreactivity which is absent from healthy brain parenchyma. The expression of both markers was clearly divergent within and between cases as shown: Cases 32 (A–C), 5 (G–I), and 23 (D–F).

In order to obtain modality fusion heatmaps for IBA1 and CD163, WSI immunohistochemical images of IBA1 and CD163 of adjacent tissue sections were converted into corresponding heatmaps. This was followed by image fusion of the respective adjacent tissue areas (Fig. 5). Fusion heatmaps allow the simultaneous visualization of both IBA1 (Fig. 5A,D,G) and CD163 (Fig. 5B,E,H) labeling in a single combined virtual WSI. The fused heat maps of IBA1 and CD163 expression confirmed areas of overlapping expression (red) and lack of such spatial correlation (white) on a per-case basis as illustrated in Fig. 5C,F,I. The results of this multimodal mapping fit well with our histopathological observations on a divergent and variable association of IBA1 positive microglia/macrophages and CD163 immunoreactive cells and with necrosis in glioblastoma.

## Discussion

Glioblastomas are distinguished by the presence of necrosis, which is a well-established diagnostic hallmark of these tumors. The presence of necrosis in glioblastomas can lead to the activation and recruitment of microglia and macrophages, which may release factors that support tumor growth, angiogenesis, and glioma invasion [17–20]. Microglia/macrophages are typically seen in large numbers within the tumor microenvironment and are considered to become subverted by glioblastoma and utilized to promote tumor growth and progression.

IBA1 and CD163 showed remarkable differences in our study in relation to glioblastoma tumor necrosis. Generally, IBA1 immunoreactive cells were far less common in necrotic tissue areas than CD163-expressing cells. We also found extensive and frequently diffuse extracellular CD163 deposition, especially in hypocellular necrobiotic tumor areas where IBA1 was typically absent. In the CNS context, IBA1 is a pan-microglia marker whereas CD163 expression is a characteristic of infiltrating macrophages but not typically found on microglia cells.

At this point a note on the differences between microglia and macrophages seems appropriate in order to prevent confusion. Microglial cells are resident macrophage precursors that reside exclusively within the central nervous system (CNS) parenchyma. They originate from erythromyeloid cells in the yolk sac early during development and possess the ability to self-renew and proliferate even in adults under pathological conditions, exhibiting remarkable phenotypic and functional plasticity. Notably, their activation into a macrophage stage is just one of many possible states, but it does not occur in normal brain tissue or under mild pathological conditions (“soft pathologies”) such as stress. Structural injury to CNS tissue caused by mechanical trauma, stroke, infections, or an expanding tumor triggers the transformation of microglia into brain macrophages. In contrast to microglia, macrophages are a broader category of cells that can arise from various sources, including bone marrow-derived monocytes, and can be found in multiple tissues throughout the body. While microglia share some functional similarities with macrophages, such as phagocytic activity and cytokine production, they possess distinct normal functions such as the interaction with synapses. Notably, microglia are the sole cell population within the CNS parenchyma that consistently expresses IBA1, a molecular feature that is conserved across species and persists at all known stages of microglial activation. An additional advantage of using IBA1 as a marker for microglia is its compatibility with paraffin-embedding, which is the most common method of storing human CNS tissue. Furthermore, there is an extensive body of literature on IBA1 compared to TMEM119, a potential alternative marker whose expression is also less reliable under disease conditions. Like IBA1, CD163 is an extremely well-established marker. Anti-CD163 antibodies label (BMDM), and there is a very high level of documentation. IBA1 (AIF1) binds actin and calcium (www.genecards.com, accessed on 12 November 2024). It consists of 147 amino acids and features EF-hand motifs. AIF1 undergoes conformational changes upon calcium binding, enabling it to function as an intracellular signaling molecule. CD163 is a member of the scavenger receptor cysteine-rich (SRCR) superfamily (www.genecards.com) and serves as an acute phase receptor for haptoglobin-hemoglobin complexes as well as bacteria and also has an anti-inflammatory role.

### Cellular expression of IBA1 and CD163

IBA1 is typically found in highly ramified microglia of the normal healthy CNS parenchyma. The expression of IBA1 increases during microglial activation in response to pathological challenges when the processes of microglial cells retract and become less ramified and stout. IBA1 expression is highest when microglia-derived brain macrophages are formed. Thus, IBA1 labeling of microglia shows a dynamic range in response to physiological and pathological tissue stimuli allowing visualization of the “microglia sensor” [21] by means of IBA1 immunohistochemical stains. This combined with the ability of anti-IBA1 antibodies to detect microglia in different species has made IBA1 a widely used anti-microglia antibody that has become a standard reference for the characterization of microglia in the CNS in both health and disease. CD163 in contrast is completely absent from normal brain parenchyma with the exception of occasional immunopositive perivascular macrophages that are normally found within the perivascular basement membrane, i.e., outside the CNS tissue proper where they undergo physiological turnover with the bone marrow via the bloodstream [22,23]. Under rare conditions that are not fully understood, CD163 may also be expressed by microglial cells (e.g., activated but still ramified microglia of the Parkinsonian substantia nigra; MBG, personal observations). Glioblastoma is another example (Fig. 4H). It is now firmly established that bone marrow-derived myeloid cells infiltrate malignant glioma tissue [24,25]. This can help explain the great inter-case and intra-tumoral heterogeneity with respect to the distribution as well as abundance of the two macrophage cell populations. Modality fusion heatmaps (Fig. 5) allowed an assessment of the distribution and colocalization of microglia and brain macrophages on a per-case basis. Necrotic tumor tissue has lost the formerly resident IBA1-positive microglia/macrophage cell population. However, macrophages that have come in via the bloodstream (e.g., Fig. 4E) populate the necrotic tissue and there is an excess of extracellular CD163 as well that may at least in part be released by these cells.

### Relevant microglia and macrophage pathways

**Figure 6:**
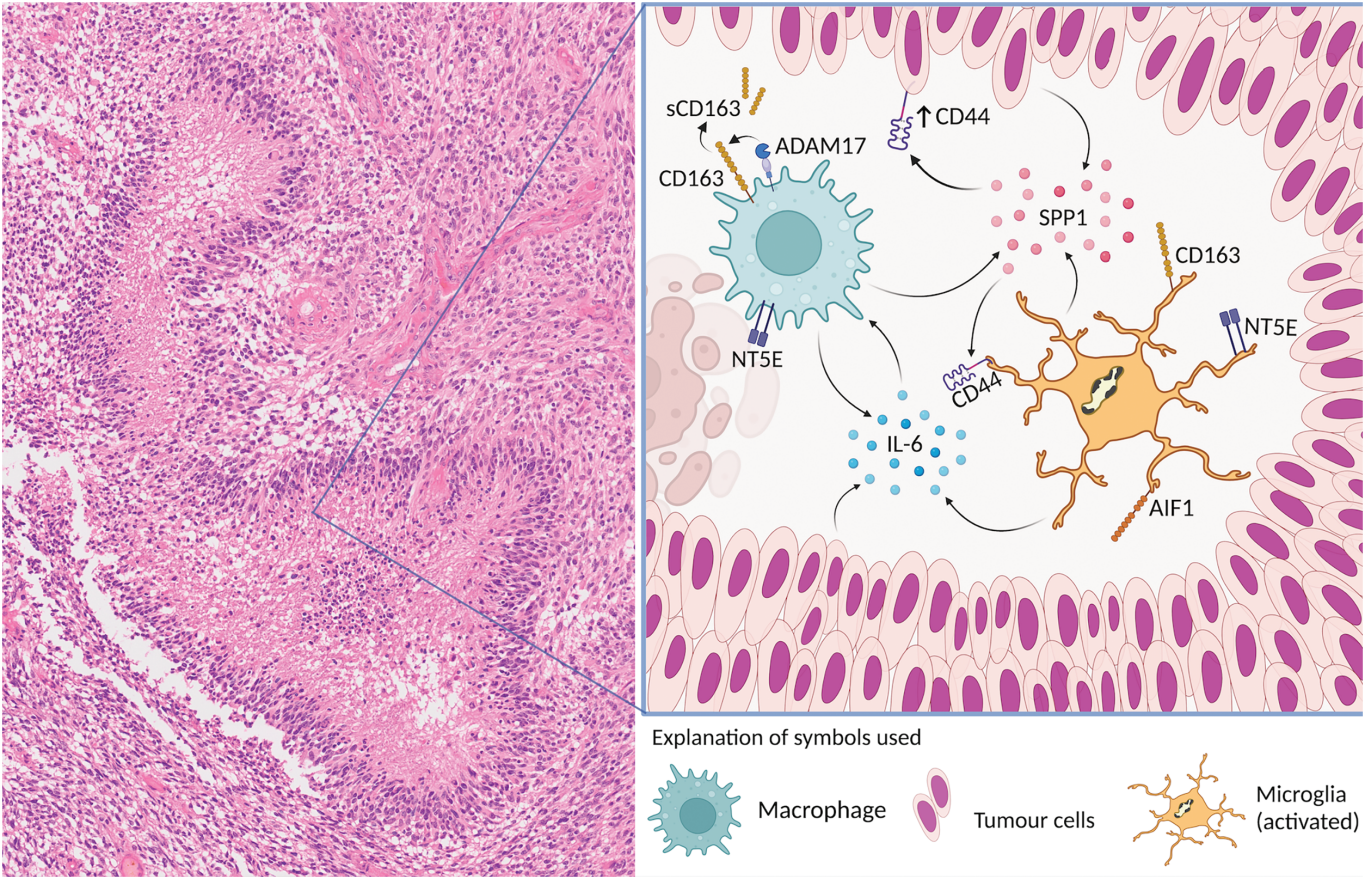
Key microglia/macrophage molecules and their interactions are schematically illustrated in relation to a typical glioblastoma necrosis. IBA1 (AIF1) and CD163 are the cellular microglia/brain macrophage markers used in this study. ADAM17 is responsible for inducing the ectodomain shedding of scavenger receptor CD163 from the membrane of activated macrophages. IL-6 is released into the hypoxic niche. IL-6 signaling may stimulate the expression of NT5E (CD73). Significant upregulation of CD44 is found in palisades of glioblastoma cells and nearby areas of necrosis. ADAM17 may also promote the cleavage of intracellular CD44. SPP1 (OPN) is a ligand of CD44 that is secreted by both myeloid and glioblastoma cells. “Created with BioRender.com (accessed on 12 November 2024)”.

Fig. 6 shows an “interactogram” for microglia/macrophage molecules that are of particular interest in the context of the present study. Soluble CD163, which has a long half-life, has been proposed as a surrogate biomarker for tumor Necrosis Factor-α (TNF-α) [26]. Increased levels of soluble CD163 can be detected in biofluids, e.g., CSF (cerebrospinal fluid), and used to monitor the activation of macrophages. ADAM17 (a disintegrin and metalloprotease 17), also known as TACE (tumor necrosis factor α-converting enzyme) is considered responsible for mediating ectodomain shedding of scavenger receptor CD163 from the membrane of activated macrophages which then becomes soluble CD163 (sCD163). It is conceivable that in the necroses of glioblastoma, ADAM17-mediated shedding of membrane CD163 from macrophages occurs which could explain the presence of the large amounts of extracellular CD163 we observed in necrotic tissue areas in this study. It is worth noting that the activation and proliferation of T-lymphocytes can be impeded by soluble CD163 [27], suggesting that soluble CD163 may also have anti-inflammatory properties and assist in the process of immune evasion of glioma cells. IL-6 is one of the molecules involved in the upregulation of CD163 expression [28]. Exosome-derived IL-6 from human glioblastoma (GBM) cells may trigger autophagy in macrophages by activating the Signal Transducer and Activator of Transcription 3 (STAT3) signaling at least *in vitro* [29]. IL-6 is released into the hypoxic niche by different cell types resulting in IL-6 downstream signaling in macrophages [28–30]. IL-6 signaling may also stimulate the expression of NT5E (CD73) [31], which is a long-known microglia activation marker [32]. Elevated expression of CD73 is observed in glioblastoma and other cancers [33]. Furthermore, significant up-regulation of CD44 is found in glioblastoma cells of pseudopalisading necrosis [34]. ADAM17 may also promote the cleavage of intracellular CD44 and enhanced stemness in glioma cells [34]. SPP1 (OPN) is a ligand of CD44 that is secreted by both myeloid and glioblastoma cells [35].

### Necrosis and microglia activation

IL-6 and CSF-1 (colony-stimulating factor-1) when present in the necrotic tumor microenvironment may induce synergistic activation of IL-6 and CSF-1 downstream pathways which promote PPARγ (peroxisome proliferator-activated receptor-γ)-dependent HIF-2α (hypoxia-inducible factor-2α) transcription resulting in a robust ARG-1 upregulation in BMDMs [28]. Increased ARG-1 expression is widely recognized as an indicator of anti-inflammatory activity in BMDMs of the glioblastoma microenvironment. It is important to remember that in a hypoxic tumor microenvironment, activation of HIF (hypoxia-inducible factor)-2α has a key role in the regulation of tumor stem cell phenotype [36]. NT5E (CD73) functions with upstream CD39 to convert extracellular ATP into adenosine [37]. In a recent experimental study, Goswami et al. reported that glioblastomas contain a subset of CD68+ macrophages that co-express NT5E (CD73) and survive anti-PD-1 treatment [38]. Further analysis revealed that macrophages expressing high levels of NT5E are characterized by elevated expression of chemokines and chemokine receptors such as CCR5, CCR2, ITGAV/ITGB5, and CSF1R, which may play a role in the recruitment of macrophages into the glioblastoma microenvironment [38]. A significant accumulation of extracellular adenosine may result in inhibitory effects on CD4+ and CD8+ T cell functions by negating signals from IL-2 receptors and TCR in T cells [39]. The results of a recent immune checkpoints correlation test by Tang et al. [33] suggest that the substantial expression of CD73 detected in various cancers is associated with several classical immune checkpoints including NRP1, CD276, and CD44. It is further worth noting that elevated levels of IL-6 and TGF-β are linked to up-regulated expression of NT5E (CD73) and CD39 in Th17 cells [40]. Again, of special relevance to necrosis, Johansson et al. [34] have shown that a high level of expression of the glioma stem cell marker CD44 is observed under hypoxic conditions.

### The molecular linkage between microglia/macrophage function, hypoxia, and tumor progression

The intracellular domain of CD44 interacts with HIF-2α promoting the hypoxic and pseudo-hypoxic phenotype of GSCs [34]. In line with findings from Johansson et al. [34], a recent study by Petterson et al. [41] has also demonstrated a significant up-regulation of CD44 in glioblastoma tumor cells, especially in perinecrotic pseudopalisades and nearby areas of necrosis. Inoue et al. have implicated CD44 in tumor recurrence in glioblastoma [42]. With respect to CD44 expression in microglial cells, Ivanova et al. [43] have discovered that deletion of CD44 in microglia hinders invasion of glioma cells. Interestingly, the membrane-bound metalloprotease, ADAM17, is capable of promoting the cleavage of intracellular CD44 and enhanced stemness in glioma cells via the activation of HIF-2α signaling [36]. The cleavage of intracellular CD44 is mediated by gamma-secretase [44]. By performing Western blot analysis, Johansson et al. [34] have observed a significant upregulation of ADAM17 mRNA levels after extended exposure to hypoxia. The authors further inhibited ADAM17 expression using TAPI-2, impeding the cleavage of CD44 into its intracellular form. Hypoxia-inducible factors (HIFs) are known to support stem-like properties in glioma cells. Johansson et al. [34] have established that the intracellular domain of stem cell marker CD44 is released under hypoxic conditions and interacts with HIF-2α (but not HIF-1α) and promotes activation of HIF targeted genes and hypoxia-induced stemness in GSCs. It is important to note that the intracellular CD44 binding by the HIF-2α-induced stem-like phenotype of glioma cells is also observed under well-vascularised conditions [45]. This may point to the possibility that the formation of tumor necrosis may not be directly induced by tumor cells outgrowing their local blood supply. Interestingly, SPP1 (secreted phosphoprotein 1) has been suggested to promote macrophage migration through the interaction with CD44 based on data from a study by Wei et al. [46]. SPP1, also known as Osteopontin (OPN), is a glycophosphoprotein expressed by various cell types, including macrophages, T-cells, and tumor cells [47]. SPP1 is able to regulate cell-matrix interactions by binding to CD44 and integrin receptors thus mediating cell adhesion, chemotaxis, angiogenesis, and resistance to apoptosis.

### CD163 and survival

**Figure 7:**
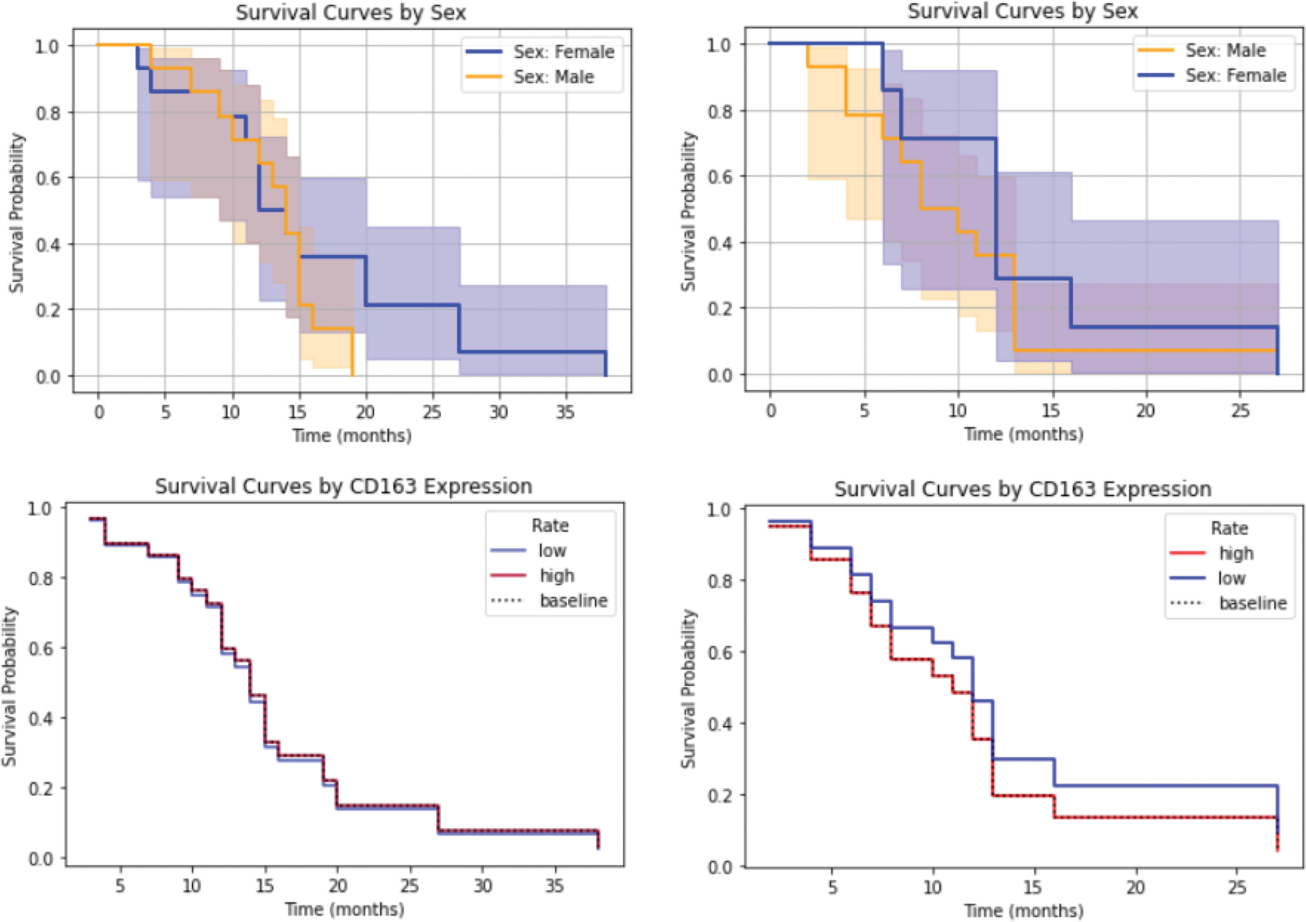
Survival analysis in relation to CD163 expression (with sex serving as a control variable). As anticipated, male GBM patients demonstrated shorter survival in both cohorts. A mild negative association between increased CD163 expression and survival was observed in the second cohort, which is discussed further in the text.

CD163 functions as a hemoglobin-haptoglobin complex receptor and is associated with the “M2” or “pro-tumor” state of macrophages whereas normal microglia do not express CD163. CD163-positive macrophages have been associated with a number of malignancies, including glioma [48]. This association was less pronounced than anticipated in our study (Fig. 7), highlighting the need for further investigation to better understand its significance. Poon et al. [49] found that there are significantly fewer microglia/macrophages in IDH-mutant high-grade gliomas and the authors hypothesized that this contributes to the better prognosis of these tumors. The latest WHO classification, which was applied to this study, excludes IDH-mutant cases from the GBM definition. Macrophage markers are currently not included in this tumor classification system.

### Limitations of this study

The human biopsies obtained from tissue repositories were collected at different hospitals, which could result in variability in sample preparation. To mitigate the impact of subjective interpretation by pathologists, all cases were reviewed by a board-certified neuropathology specialist. However, immunohistochemical studies have additional limitations. Immunohistochemistry (IHC) is susceptible to variations in antibody specificity and sensitivity, as well as technical factors such as fixation methods and antigen retrieval processes. Nevertheless, we have extensive experience with the markers employed in this study, particularly IBA1, which has also been used in our experimental animal studies that include work done in the laboratory where the IBA1 antibody was originally developed and validated.

## Conclusions

One striking finding of this study is that IBA1 and CD163 labeling, and by inference microglial cells and bone marrow-derived brain macrophages, exhibit a divergent and variable association with necrosis in glioblastoma. Furthermore, the histological picture of IBA1 and CD163 immunostains in glioblastoma is complex also in non-necrotic areas. Since we have been able to make use of adjacent tissue sections, it was possible to see that even in instances where IBA1 labeled round macrophages, the corresponding labeling for CD163 did not necessarily indicate an accumulation in the same tissue area in all cases. Thus, IBA1 immunoreactive macrophages in glioblastoma tumor tissue likely represent brain macrophages that are derived from resident microglia. Digital image analysis (multimodal fusion maps of WSIs) proved invaluable for confirmation of the microscopic results. The high number of myeloid cells in glioblastoma and the fact that their presence is a constant feature of the glioma tumor microenvironment along with emerging evidence that links tumor-associated macrophages (TAMs) to tumor progression makes this analysis quite important.

The observed differential expression of IBA1 and CD163 may indicate that microglia and myeloid macrophages have different functions within the tumor microenvironment, or it could suggest that other factors are influencing their distribution and relative abundance. Our observation that local microglia are more likely to be involved in diffuse infiltration of glioma cells in hypercellular tissue areas, while myeloid macrophages dominate in necrotic regions, suggests that targeting specific macrophage populations may have implications for effective treatment. For instance, therapies aimed at modulating microglial activity may be more beneficial in reducing diffuse infiltration, whereas the targeting of myeloid macrophages may be more effective where necrosis has occurred and these cells predominate.

The likely different roles of microglia, microglia-derived brain macrophages, and (BMDM) in the tumor microenvironment of glioblastoma are particularly important for immunotherapeutic approaches. A deeper understanding of the interactions between microglia, brain macrophages of both origins and glioblastoma stem cells seems foundational for the development of novel therapeutic approaches that can more effectively target this highly malignant disease.

## Data Availability

The datasets generated and/or analyzed during the current study are available from the corresponding author on reasonable request.

## Appendix A

**Table A1:** Characteristics of glioblastoma cases

Case	Sex	Age at diagnosis	Survival (months)	Localisation
1	F	62	16	Right occipital
2	M	55	14	Left occipital, parietal
3	F	46	10	Left frontal
4	M	70	4	Right frontal
5	F	54	95	Left parietal
6	M	33	15	Right frontal
7	F	57	4	Right frontotemporal
8	F	48	27	Right frontal, occipital, parietal, temporal
9	M	65	14	Right frontal
10	M	69	16	Right frontal
11	M	51	23	Left parietal
12	F	55	12	Left frontal
13	F	85	4	Left temporal
14	F	72	13	Right temporal
15	M	77	20	Right parietal
16^1^	M	33	15	Right frontal
17	F	51	Unknown	Right frontal
18	M	50	16	Left temporal
19	F	60	20	Right temporal
20	F	75	27	Right frontal
21	M	65	10	Left temporal
22	M	33	8	Right frontal
23	F	60	21	Left temporal
24	M	68	10	Left frontal
25	F	59	38	Right occipital
26	F	79	12	Right parietal
27	M	73	17	Right frontal
28	M	50	20	Right parietal
29	M	66	12	Right parietal
30	M	50	15	Right temporal
31	M	78	5	Right frontal
32	F	60	15	Right occipital
33	M	75	16	Right occipital
34	F	62	13	Right occipital, parietal

Note: ^1^Same as Case 6 (subsequent biopsy).

**Table A2:** Characteristics of glioblastoma cases

Case	Sex	Age at diagnosis	Survival (months)	Localisation
1	M	62	5	Right frontal
2	M	72	13	Left temporal
3	M	77	3	Right frontal
4	F	56	16	Midline frontal
5	M	59	10	Left parietooccipital
6	F	57	7	Right frontal
7	M	71	6	Right temporal
8	M	77	8	Right temporal
9	F	63	3	Right parietooccipital
10	M	68	9	Right frontal
11	F	75	7	Right occipital
12	M	61	13	Left parietal
13	M	68	9	Right frontal
14	M	42	14	Right temporal
15	F	71	9	Left temporooccipital
16	M	41	13	Left parietal
17	F	76	13	Right frontotemporal
18	F	59	12	Right occipital and parietal
19	M	84	11	Left occipital
20	M	34	27	Left medial temporal
21	M	81	5	Left parietal
22	M	74	7	Left parietal
23	F	74	6	Left temporal
24	F	47	12	Left temporal
25	F	62	27	Right frontal
